# Application of Low-Cost Methodologies for Mobile Phone App Development

**DOI:** 10.2196/mhealth.3549

**Published:** 2014-12-09

**Authors:** Melvyn Zhang, Enquan Cheow, Cyrus SH Ho, Beng Yeong Ng, Roger Ho, Christopher Cheng Soon Cheok

**Affiliations:** ^1^National HealthCare GroupSingaporeSingapore; ^2^Department of Community PsychiatryInstitute of Mental Health SingaporeSingaporeSingapore; ^3^Department of Psychological MedicineNational University HealthCare SystemsSingaporeSingapore; ^4^Department of PsychiatrySingapore General HospitalSingaporeSingapore; ^5^National Addictions Management ServiceInstitute of Mental HealthSingaporeSingapore

**Keywords:** education, technology, mobile phone apps, cost-effectiveness

## Abstract

**Background:**

The usage of mobile phones and mobile phone apps in the recent decade has indeed become more prevalent. Previous research has highlighted a method of using just the Internet browser and a text editor to create an app, but this does not eliminate the challenges faced by clinicians. More recently, two methodologies of app development have been shared, but there has not been any disclosures pertaining to the costs involved. In addition, limitations such as the distribution and dissemination of the apps have not been addressed.

**Objective:**

The aims of this research article are to: (1) highlight a low-cost methodology that clinicians without technical knowledge could use to develop educational apps; (2) clarify the respective costs involved in the process of development; (3) illustrate how limitations pertaining to dissemination could be addressed; and (4) to report initial utilization data of the apps and to share initial users’ self-rated perception of the apps.

**Methods:**

In this study, we will present two techniques of how to create a mobile app using two of the well-established online mobile app building websites. The costs of development are specified and the methodology of dissemination of the apps will be shared. The application of the low-cost methodologies in the creation of the “Mastering Psychiatry” app for undergraduates and “Déjà vu” app for postgraduates will be discussed. A questionnaire survey has been administered to undergraduate students collating their perceptions towards the app.

**Results:**

For the Mastering Psychiatry app, a cumulative total of 722 users have used the mobile app since inception, based on our analytics. For the Déjà vu app, there has been a cumulative total of 154 downloads since inception. The utilization data demonstrated the receptiveness towards these apps, and this is reinforced by the positive perceptions undergraduate students (n=185) had towards the low-cost self-developed apps.

**Conclusions:**

This is one of the few studies that have demonstrated the low-cost methodologies of app development; as well as student and trainee receptivity toward self-created Web-based mobile phone apps. The results obtained have demonstrated that these Web-based low-cost apps are applicable in the real life, and suggest that the methodologies shared in this research paper might be of benefit for other specialities and disciplines.

## Introduction

The worldwide prevalence of mobile phones has made them a very useful and innovative platform for the provision of patient care [1], as well as in helping clinicians with management decisions. Over the past few years, mobile phones have advanced drastically in terms of both their functionality and design, and they are currently more than what used to be a simple call and messaging device [1]. They have literally been transformed into sophisticated personal mini-computers.

Previous reviews have highlighted the worldwide prevalence and the global acceptance of mobile phones and commercial mobile apps by medical students, trainees, and interns. In 2012, a questionnaire-based survey was distributed amongst interns in the Republic of Ireland [2], and it has demonstrated that mobile phones are being used daily by the interns to perform their job. There have been numerous literatures demonstrating the acceptability of mobile phone usage in education for medical students. A survey questionnaire conducted at the University of Birmingham, United Kingdom, highlighted that students generally find mobile phones useful as educational aids, with at least 84% believing so. This has been replicated in other studies, which highlighted that there has been a high level of mobile phone ownership and usage amongst medical students and junior doctors [3].

Along with the enhancements in mobile phone functionality, there has been an increased number of educational mobile phone apps made available for users to download and install. However, it is a well-known fact that anyone could publish a medical app, and the app stores do not routinely do a rigorous review of the accuracy of the content of the mobile app prior to publication [4]. Although mobile phones have been used by the majority of interns on a daily basis in performing their job [4], there still needs to be more guidance and advice with regards to the accuracy and the credibility of the information provided within the apps [5]. Despite the high usage of mobile phone and its apps, the development of mobile phone apps by professionals will incur a huge cost. Very often, clinicians and researchers have to wait for and hope to be successful in securing grants to finance the developmental costs. Apart from the concerns about the high cost associated with development, another concern lies with the fact pertaining to how evidence-based apps are. Most of the current apps available have been developed by external vendors and developers, and are lacking inputs from clinicians who have a vast amount of knowledge and expertise in their specialized fields. Recent studies have highlighted the need for clinicians to be more involved in the mobile phone app development process and a research article highlighted a simple methodology of creating an application using just an Internet browser and a text editor [6]. The methodology shared seemingly seemed to help overcome the fears of clinicians, but the methodology shared previously does require clinicians to have some fundamental technological knowledge. Also, the previous methodology shared does not enable clinicians to include more multimedia features in the app. Zhang MWB et al [7] recently shared two methodologies of app development using a blogging site as well as an online app builder. The limitation of Zhang MWB et al [7] recent publication lies within the fact that the costs associated with using these tools have not been explicitly declared. In addition, the methodology shared by Zhang MWB et al [7] will still require users to have constant access to the Internet in order to access the apps. As the apps are not in the respective app stores, it might be hard for dissemination of the apps as well.

Thus, taking these variables into consideration, we wish to elaborate on the previous methodologies shared by Zhang MWB et al [7]. Therefore, the objectives of the current research article are to: (1) highlight a low-cost methodology that clinicians without technical knowledge could use to develop educational apps; (2) clarify the respective costs involved in the process of development; (3) illustrate how limitations pertaining to dissemination could be addressed; and (4) to report initial utilization data of the apps and to share initial users’ self-rated perception of the apps.

## Methods

### Low-Cost Mobile Phone App Development

Zhang MWB et al [7] previously described two methodologies that could be deployed for mobile app creation. In this paper, we wish to highlight a particular methodology that could enable clinicians to develop mobile apps that resemble native mobile phone apps developed by professionals, with minimum costs involved.

**Figure 1:**
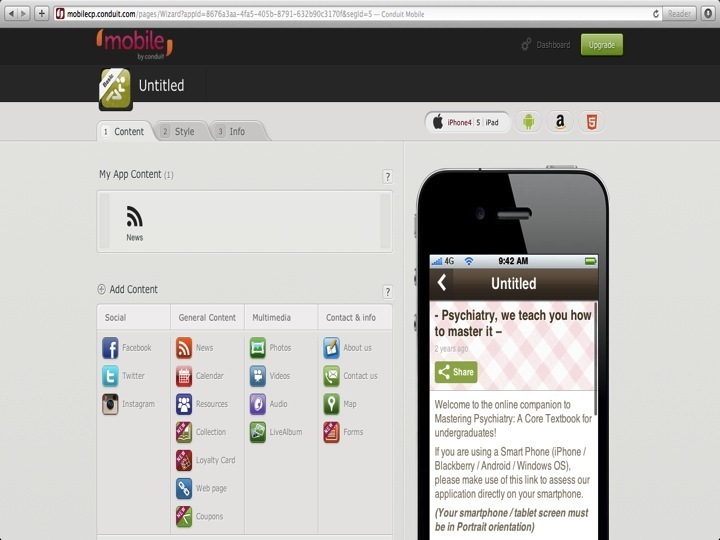
Development of Web-based mobile phone using online app builder.

**Figure 2:**
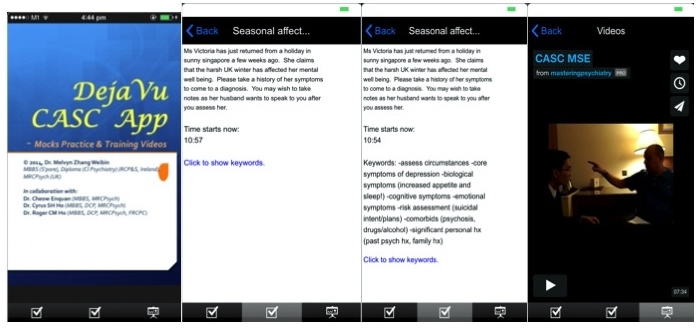
Overview of the app.

There are various online Web-based mobile phone app builders such as Conduit Mobile [8] and IbuildApp [9]. The advantages of using online Web-based mobile app builders are that its graphic user interface will help in the immediate integration of text-based content, videos, questionnaires, and other multimedia features. These multimedia features include built-in photo-taking capabilities as well as e-commerce capabilities. An overview of the features that could be integrated using an online application builder is exemplified (Figures 1 and 2). Integration of content was simple. All users have had to do is select the appropriate interactive feature and then key in the relevant information.

The methodology described above would enable clinicians to devise cost-effective apps. A summary of the costs involved and the features included for each price plan provided by these online app builders have been summarized in Table 1. Mobile site visits imply that users could access the application via a Web-link.

The authors will describe the development of two educational apps, one for undergraduate education and another for postgraduate education using the cost-effective methodologies as described above.

**Table 1:** Cost of online mobile app builders.

Online app builder	Cost involved	Features
Conduit Mobile	US $0 /month	up to 5 mobile app downloads
		50 mobile site visits per month
Conduit Mobile	US $33/month	unlimited app downloads
		unlimited mobile site visits
		app submission to stores
IBuildApp	US $5.99/month	100 app downloads
		unlimited mobile site visits
IBuildApp	US $23.40/month	3000 app downloads
		unlimited mobile site visits
		expert submission to app stores

### Differentiating “Mastering Psychiatry” and the “Déjà vu” Apps

#### The “Mastering Psychiatry” Mobile Phone App

Zhang MWB et al [7] has previously described the application of this methodology in their recent paper. An online mobile app builder was used to develop the initial version of the app, including various features such as the core textbook information, clinical interviewing videos, and questionnaires. All the information was coded into the online builder using basic HTML5 programming language. The determination of the relevant content to be included was based on the experiences of the authors in undergraduate education, and also based on careful evaluation of the existing textbook based mobile app in the mobile app stores. With regards to the dissemination of the mobile app information, students who posted to the National University of Singapore, Psychological Medicine program were provided with information of the mobile app on the first day of their clinical posting.

#### The “Déjà vu” Mobile Phone App

The “Déjà vu” CASC (Clinical Assessment of Skills and Competencies) app was developed between January 2014 and April 2014. The developmental approach involved 4 developmental phases, which included: (1) understanding trainee requirements; (2) system development; (3) system evaluation; and (4) system deployment. In terms of user requirements, the authors postulate that the core requirements of a CASC app for trainees would need to include each one of the following in Textbox 1.

The postulation of what to be included in the mobile app was based on the experiences of the authors in the recent diet of the postgraduate examination.

In consultation with the author RCMH, the following core interviewing approaches have also been identified to be essential as presented in Textbox 2.

**Core requirements for trainees.**

1. Inclusion of Mock examination stations with timers (either 7 minutes or 10 minutes timers).
2. Timers to include additional 60 seconds preparation time, to allow trainees to practice recalling vital information and write them down prior to the commencement of any station.
3. Inclusion of mock stations (30 in total) that are adaptations of old College stations with variants in the constructs, or are new stations crafted based on information from the Royal College of Psychiatrists Mental Health Leaflets.
4. Ability of app to enable trainees to link up with others for video-conferencing.
5. Ability of app to enable trainees to practice stations in a timed mock examination way with fellow trainees from their trust.
6. Inclusion of instructional videos that demonstrate to trainees core approaches for a specific variety of stations.

**Core interviewing approaches.**

1. How to break bad news
2. How to deal with angry patients
3. How to perform a mental state examination
4. How to handle an explanation station
5. How to deal with disinhibited patients
6. How to deal with patients who are refusing to engage with Psychiatry
7. How to perform a risk assessment
8. How to deal with patients who are not forthcoming
9. How to handle patients with learning disabilities
10. How to perform cognitive assessment

With regards to system deployment, this was initially an issue as none of the authors are currently based in the United Kingdom, and hence, would not have access to the trainees within each of the trusts. Therefore, the deployment of the mobile phone app was done via a commonly accessed online forum, known as “Revise Now”. The author, MWBZ, posted a message under the forum topic ‘courses and books’ on April 13^th^ 2014, giving trainees a Web-link to access the mobile phone app. In addition, the mobile phone app was also deployed locally in Singapore to fellow trainees via direct email dissemination.

Subsequently, as the online app builder allows for the generation of an application programming interface (API) for download, the author, MWBZ managed to submit a version of the mobile app to the Android Play Store and has been published since October 27^th^, 2014. In order to be published on the mobile app stores, MWBZ has had to set up a developer account and upload the API, along with images of the app. Publication to the iTunes store was similar, though the cost of the developer account was vastly different. The costs were US $25 per annum for an account with Android Play, but US $300 per annum for an account with the iTunes store.

The application could be found in the android play store by searching for the following keywords “MRCPsych, CASC.”

### Acquiring Utilization Data and Initial Perspectives

Utilization data was tracked using the analytics provided by the online app builders. With regards to initial perspectives, the authors, with ethics approval from the National University of Singapore, conducted a user-perspective survey looking into the perception of the mobile app, mainly by undergraduate students. Participation in the survey was entirely voluntary and a relevant participant information handout was provided to all the participants prior to the start of the survey.

## Results

### The “Mastering Psychiatry” Mobile App

The online portal and the Web-based mobile phone app were launched on July 15^th^ 2012 via direct dissemination of the Web-links of the mobile app. Based on our analysis, since inception and up until November 7^th^ 2014, a cumulative total of 722 users have used the mobile app.

### The “Déjà vu CASC” Mobile App

Since the date of deployment of the Déjà vu CASC mobile app and up until November 7^th^ 2014, there has been a cumulative total of 154 downloads of the mobile app from the Web-link that was provided to the students.

For the user’ perceptive survey about the “Mastering Psychiatry” app launched locally in Singapore, a total of 185 undergraduates have taken part in the survey. Most of them used an Apple IOS device (121/227, 53.3%), whereas 21.6% (49/227) used an android device. The average age of the sample was 22 (141/178, 79.2%). A cumulative total of 51.7% perceived that the mobile app to augment undergraduate education is helpful. Approximately 71 of the students agreed that mobile apps would make a good companion to a conventional textbook.

## Discussion

### Principal Findings

From our current knowledge, this is one of the first few studies to describe how using an online application builder might be a low-cost methodology of mobile app development, with interactive features embedded within the app. Based on the utilization data for both the undergraduate and the postgraduate apps, it showed that both groups are receptive towards a self-developed mobile app. Therefore, our current study has demonstrated that clinically relevant content for mobile phones could be developed by clinicians and clinical teachers using low-cost, non-technical methodologies. The results obtained indicate that these Web applications could be used in real-life and hence may suggest that these low-cost methodologies are feasible for conveying knowledge to other health professionals.

Despite the rapid advancement in Web 2.0 technologies as well as mobile phone technologies, there is still a paucity of technology-related papers published about how lost-cost methodologies could be used by clinicians to self-create mobile phone apps. The only study identified thus far is previously published by Waldmann UM and Weckbecker K (2013) [10], who have described how they have formulated a Web-based guideline app and have piloted it and demonstrated that students were receptive towards such a self-developed app. Zhang MWB et al [7] have described two separate methodologies that clinicians could use to develop mobile apps, but have not disclosed the costs involved. It is hoped that this current paper will share more insights about the relevant costs involved. Also, the limitations of Zhang MWB et al’s [7] previous paper was that there was perceived difficulties in accessing the mobile apps, as they are not made available on the mobile app store. In our current paper, the authors have shared how the respective online app builders could help to make the mobile app available on the respective app stores. Zhang MWB et al [7] also mentioned that their findings are only generalized to their local context of students in Singapore. In the current paper, by using a low-cost method to create a postgraduate application, the authors have managed to demonstrate the utility of a self-created mobile app worldwide.

The authors in this paper have described a low-cost methodology that could enable clinicians to develop their own in-house mobile app. This might help mitigate the concerns raised by Thomas LL (2013) [11] in his previous editorial reply, which proposed that there ought to be a systematic self-certification model developed for peer-review of mobile apps. By empowering clinicians with techniques of mobile app development, and hopefully enticing them with the low-costs associated with development, more apps made available on the application would fulfill the quality of information based on Health on the Net Foundation (HON) criteria. The criteria specifies that all relevant medical information included in any mobile app in the application stores should be attributed to an author, with the training level of the author disclosed. In addition, the purpose of the mobile app, confidentiality of the information, date of the information as being created and modified, and the contact details and disclosures should all be specified within the application.

The main strength of the current study is that we managed to demonstrate how educational mobile apps could be developed using low-cost methodologies and without any technical knowledge. It is obvious that if clinicians take more ownership of creating mobile apps, the app will be more evidence based and there will be enhanced quality of information within the app. Our current study has empowered both undergraduates and postgraduate psychiatry students and trainees to have an opportunity to make use of the latest innovations in technologies, using a low-cost methodology. As well, the initial results also demonstrate the feasibility of adopting this methodology in creation of mobile apps for education and for other disciplines. In addition, the provision of the application on the app store would imply that users do not need a continuous Internet to access the relevant content.

Nevertheless, several limitations remain in the current study. The authors have not formally evaluated the perspectives of non-Asian users of self-created mobile apps. The authors have addressed the previous limitations pertaining to the difficulties associated with the access of the mobile app, by making the app available on one of the app stores. However, the authors have not mentioned their experience with making the mobile app available on other stores, such as iTunes. It should be noted that different app stores have different criteria for acceptance of mobile apps and there might be a chance one store would accept and another might reject the proposal. This would limit the distribution of the mobile app across multiple computing platforms. Another limitation would be the lack of conducting focus groups before deciding what would be the most appropriate contents to be integrated within the respective mobile apps.

### Conclusions

This is one of the few studies that has demonstrated that clinically relevant content for mobile phones could be developed by clinicians and clinical teachers using both low-cost, and non-technical methodologies. The results obtained have demonstrated that these Web-based low-cost mobile apps are applicable in real life, and suggest that the methodologies shared in this research paper might be of benefit for other specialties and disciplines. In addition, it is hoped that more clinicians will be willing to consider using our methodology and create their own mobile apps, and by pooling the results of several studies together, we will have more rigorous evidence of the effectiveness of self-created mobile phone apps.

## Abbreviations

API: application programming interface
CASC: Clinical Assessment of Skills and Competencies
